# Competitive Immunoassay of Melatonin by QCM Biosensor

**DOI:** 10.3390/bios16080399

**Published:** 2026-07-23

**Authors:** Miroslav Pohanka

**Affiliations:** Military Faculty of Medicine, University of Defense, Trebesska 1575, CZ-50001 Hradec Kralove, Czech Republic; miroslav.pohanka@unob.cz

**Keywords:** antioxidant, biosensor, blood, competitive assay, circadian rhythm, ELISA, piezoelectricity, quartz crystal microbalance, saliva

## Abstract

Melatonin is a hormone that plays a role in the regulation of circadian rhythms and is also recognized for its antioxidant and cytoprotective functions. Its determination in biological samples is analytically challenging because the physiological concentration is very low, especially in saliva. Here, a competitive immunoassay based on a quartz crystal microbalance (QCM) biosensor was developed for melatonin determination. The biosensor employed a surface modified with a melatonin–albumin conjugate and a multilayer antibody amplification scheme to enhance the piezoelectric response. The assay was calibrated in the low picogram per liter range and provided a limit of detection of 0.55 pg/mL (2.37 pmol/mL) for a 25 µL sample. The analytical performance was sufficient for the determination of physiological melatonin concentrations in saliva and blood. The assay showed satisfactory selectivity against selected potential interferents and correlated strongly with a standard ELISA method, with a coefficient of determination of r^2^ = 0.994. Application to authentic human saliva samples confirmed the practical utility of the method, and the prepared biosensors remained stable for at least 140 days. The proposed QCM immunoassay represents a simple and sensitive alternative to conventional melatonin assays and demonstrates the potential of piezoelectric biosensing for non-invasive hormone monitoring.

## 1. Introduction

Melatonin is an indoleamine hormone derived from tryptophan that is synthesized mainly in the pineal gland and released in a circadian pattern with nocturnal predominance [1,2]. Its primary physiological function lies in coordinating the sleep–wake rhythm and stabilizing internal biological timing, but the molecule also exerts a broad spectrum of antioxidant and cytoprotective effects [3,4,5,6]. Melatonin acts mainly through MT1 and MT2 receptors, which are high-affinity G protein-coupled membrane receptors encoded by the MTNR1A and MTNR1B genes [7,8]. They are especially involved in circadian regulation, sleep–wake timing, neuroendocrine control, retinal physiology, vascular responses, immune modulation, and reproductive functions. Both receptors occur in the central nervous system, particularly in regions related to biological timing, and they are also present in several peripheral tissues, including the retina, cardiovascular system, immune cells, reproductive organs, skin, gastrointestinal tract, liver, kidney, and endocrine tissues. A third melatonin-binding site, historically termed MT3, has been linked mainly to quinone reductase 2/NQO2 and should therefore be distinguished from the classical MT1 and MT2 membrane receptors [9,10]. Other intracellular or nuclear melatonin-related targets have been proposed, but MT1 and MT2 remain the principal recognized mammalian melatonin receptor subtypes.

Melatonin can scavenge reactive oxygen and nitrogen species, mitigate oxidative stress in neural and peripheral tissues, and support endogenous antioxidant systems [11,12,13]. In human blood, melatonin concentrations fluctuate from low picogram per milliliter to low nanogram per milliliter levels depending on the time of day, whereas salivary concentrations are typically an order of magnitude lower [14,15,16,17]. Despite the low abundance, both matrices reflect endogenous secretion reliably, which makes melatonin an attractive biochemical marker for the assessment of circadian physiology and disorders.

A variety of analytical strategies have been introduced for melatonin determination. Immunochemical assays, including the enzyme-linked immunosorbent assay (ELISA), remain popular owing to their operational simplicity and compatibility with routine clinical workflows [18,19,20]. Because melatonin is a low-molecular-weight molecule with only one main antibody-binding epitope, it cannot be efficiently determined via a conventional sandwich ELISA format; therefore, melatonin immunoassays are usually arranged as competitive assays in which free melatonin in the sample competes with an immobilized or labeled melatonin conjugate for binding to specific antibodies.

Chromatographic methods coupled with fluorescence, electrochemical, or mass spectrometric detection provide excellent analytical performance but require specialized instrumentation [21,22,23,24]. However, these instrumental approaches are relatively expensive and require trained personnel for sample preparation, operation, and data interpretation, which limits their routine practical use and usually confines melatonin analyses to specialized laboratories, such as central hospital laboratories or regional diagnostic facilities. A major obstacle in melatonin analysis arises from its very low physiological concentration, particularly in saliva, where non-invasive sampling is highly desirable for both clinical applications and field studies. The convenience of saliva collection contrasts with the extremely low melatonin content, placing stringent demands on the sensitivity, selectivity, and background suppression of the employed analytical methods.

Biosensor-based platforms have gained attention as alternatives to conventional laboratory assays because they enable rapid, miniaturized, and potentially point-of-care measurements. One group of such biosensor platforms is represented by piezoelectric biosensors, which convert events occurring on a modified sensing surface into measurable mechanical responses, most commonly shifts in resonance frequency [25,26]. Their signal is related to mass loading, viscoelastic changes, or binding interactions at the interface between the recognition layer and the sample. This principle enables label-free monitoring of biomolecular interactions and is suitable for constructing compact analytical devices. Within piezoelectric biosensors, quartz crystal microbalance (QCM) biosensors represent a mechanical transduction approach that quantifies molecular interactions via frequency shifts induced by mass and viscosity changes at the sensor surface [27,28]. QCM devices offer several practical advantages: they operate label-free, require minimal sample processing, and can be manufactured in compact formats. These attributes make QCM technology a promising replacement or complement to optical biosensing principles, which often depend on complex optics or fluorescence labels. Piezoelectric biosensors for melatonin have not commonly been considered in previous studies, and no fully developed assay of this type has been reported; however, other biosensor formats, especially electrochemical and optical platforms, have been described and represent functional alternatives to standard instrumental methods [29,30,31,32,33].

The present study describes the development of a QCM-based immunosensor tailored for the sensitive detection of melatonin. The biosensor employs a surface-immobilized antibody together with a mass-enhancing strategy to achieve detection limits suitable for salivary melatonin, thereby enabling non-invasive assessment of circadian hormone levels. The work focuses on sensor fabrication, analytical performance, and the feasibility of using QCM technology as a practical platform for melatonin monitoring.

## 2. Materials and Methods

### 2.1. QCM Biosensor Preparation

The study utilized QCM resonators (Krystaly a.s., Hradec Kralove, Czech Republic) with a quartz thickness of 0.166 mm and a quartz disc diameter of 20 mm, featuring disc-shaped gold electrodes (7 mm in diameter) on opposite sides. The new QCMs had a basic oscillation frequency of 10 MHz prior to further processing. Before use, the QCMs were washed with deionized water and 96% (*v*/*v*) ethanol. After drying, 50 µL of 50 mg/mL cysteamine was spread on the gold electrodes and allowed to chemically activate in a wet chamber for 5 h. In the next step, unreacted cysteamine was removed, and the electrodes were washed twice with 50 µL of deionized water (hereafter referred to as washing with deionized water). Then, 50 µL of 5% (*w*/*w*) glutaraldehyde was applied to the electrodes in a wet chamber for another 5 h, followed by washing with deionized water. In the final step, a melatonin conjugate with bovine serum albumin (Cloud-Clone Corp., Wuhan, China), dissolved in phosphate-buffered saline (PBS) to a concentration of 1 mg/mL, was spread over one electrode in a volume of 50 µL and incubated in a wet chamber for another 5 h. The prepared biosensors were washed with 50 µL of PBS containing 0.1% (*w*/*w*) Tween-20 (hereafter referred to as washing with PBS-Tween) and 50 µL of PBS (hereafter referred to as washing with PBS). The immobilization of the melatonin conjugate was repeated twice, each time followed by washing with PBS-Tween and PBS. The fully prepared biosensors were then dried and stored at 4 °C in the dark until further use.

### 2.2. Melatonin Assay Using a Biosensor

**Figure 1:**
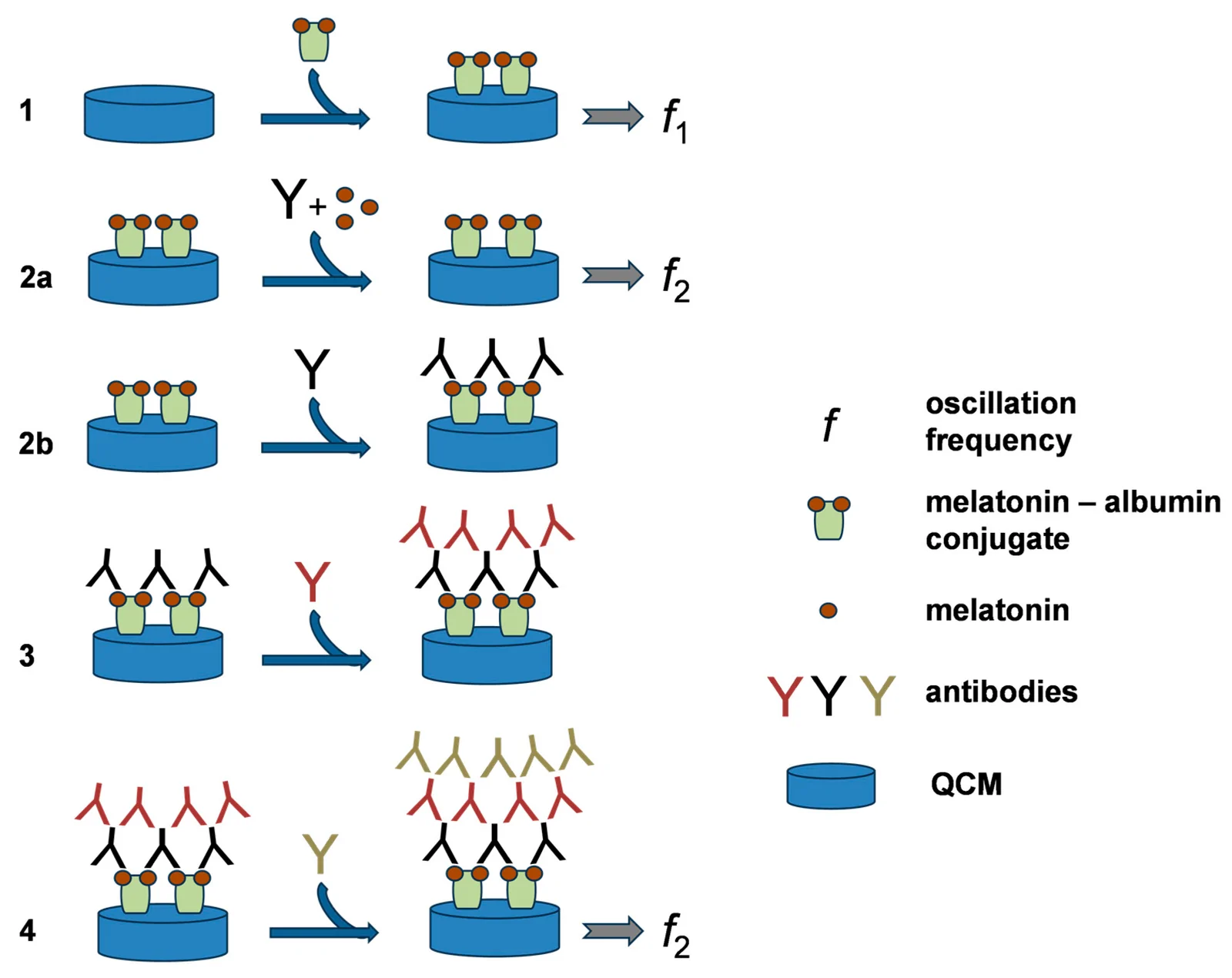
Principle of a competitive melatonin immunoassay using a QCM biosensor. (**1**) Biosensor preparation by immobilization of a melatonin–albumin conjugate. (**2a**) Competitive step—when melatonin is present in the sample, the primary antibody is blocked and does not bind to the surface; antibody layers formed under this condition are not shown. (**2b**) In the absence of melatonin, the primary antibody binds to the immobilized conjugate. (**3**) Binding of the secondary antibody. (**4**) Binding of the tertiary antibody. The oscillation frequency is measured before the assay (f1) and after the assay (f2).

Oscillation frequencies of the biosensors were measured using an ICM Level Oscillator 10.000 MHz (ICM, Oklahoma City, OK, USA) and a UZ 2400 frequency counter (Grundig, Nuremberg, Germany). All oscillation frequency measurements were performed with dried biosensors to avoid the influence of variable viscosity in different samples. First, the frequency of a new biosensor (f1) was recorded, and the immunochemical part of the assay was then performed. A 25 µL sample was mixed with 25 µL of anti-melatonin rabbit recombinant monoclonal IgG antibody (Abcam, Cambridge, UK) diluted 1:1000 in PBS, preincubated for 30 min, and applied to the electrode of the QCM biosensor. After incubation for 30 min, the biosensors were washed with PBS-Tween and PBS. In the second step, anti-rabbit IgG monoclonal antibody (mouse IgG isotype; Abcam), diluted 1:1000, was applied in a volume of 50 µL for 30 min, and the electrodes were washed again with PBS-Tween and PBS. Finally, goat-produced polyclonal anti-mouse IgG antibody (Merck; Rahway, NJ, USA), diluted 1:1000 in PBS, was applied in a volume of 50 µL per electrode for 30 min and then washed with PBS-Tween and deionized water. Finally, the biosensor was dried under laboratory conditions. Each biosensor was used only once for a single assay and was therefore treated as a disposable analytical device. All assays were performed in a laboratory under standard ambient temperature and pressure (SATP) conditions. The oscillation frequency (f2) was measured. The principle of the melatonin assay is depicted in Figure 1.

### 2.3. Samples

Melatonin was used as a standard, and acetylsalicylic acid, caffeine, 5-methoxytryptamine, and tryptamine were tested as potential interferents. Melatonin and the lipophilic interferents were first dissolved in 96% (*v*/*v*) ethanol and then diluted in PBS so that the final ethanol concentration remained below 1%. The compounds were purchased from Merck. All reagents were of p.a. (pro analysis) grade and were dissolved in PBS. Human saliva collected in the morning served as the real sample matrix. Saliva was selected as the crucial sample matrix for biosensor-based melatonin analysis because it enables non-invasive monitoring of this hormone, whereas other types of human biological samples could not be included due to practical and ethical obstacles associated with their acquisition. The saliva samples were stored frozen at temperatures below −20 °C until analysis, which was performed after approximately one month.

### 2.4. Standard Assay via ELISA

A commercially available ELISA kit (Abcam) was employed as the reference method for validation and verification of the concentrations obtained by the QCM biosensor, thereby supporting the conclusions derived from the piezoelectric QCM biosensor measurements. The assay was carried out using the standard ELISA kit components and protocol exactly as specified by the manufacturer, including reagent preparation, incubation and washing steps, and absorbance reading. Results were calculated from the kit calibration curve and used for method comparison.

### 2.5. Data Processing

The change in frequency during the assay was calculated as the difference between f1 and f2, according to the formula Δf = f1 − f2. All samples were measured five times, and the standard deviation was calculated from the replicate values. The limit of detection was estimated using the signal-to-noise approach with the conventional S/N = 3 criterion [34]. In this approach, the lowest reliably detectable concentration is defined as the analyte level producing an analytical signal three times higher than the background noise; repeated measurement of PBS was used to estimate this noise. Interference testing was evaluated using one-way ANOVA with significance levels of *p* < 0.05 and *p* < 0.01. Calibration plots were processed using OriginPro 8.5.0. (Northampton, MA, USA; https://www.originlab.com/). Extrapolation of the calibration curve was performed using the Hill 1 equation in Origin software.

## 3. Results

The QCM biosensors were prepared as described in the Section 2. The aim was to construct a simple analytical tool that employs principles typical of conventional immunoassays, such as ELISA, while using the QCM platform as the transduction system. The prepared biosensors were proven to be functional in the experiments described below, and both chemical standards and real biological samples were selected for analytical testing. The antibodies used for biosensor construction were diluted to concentrations that still provided a high analytical signal while keeping nonspecific interactions low.

The biosensor assay was based on three sequential antibody applications. Only the first antibody application contained the sample, in which free melatonin competed with the immobilized melatonin–albumin conjugate for binding to the primary anti-melatonin antibody. The two subsequent antibody applications served as signal-amplification steps and were necessary to obtain sufficiently low limits of detection for measuring physiological concentrations of melatonin in saliva.

The total time required for biosensor preparation was calculated from the individual incubation steps used during surface activation and immobilization of the melatonin–albumin conjugate. When only the defined incubation periods are considered, and processing steps such as washing, pipetting, and drying are not included, biosensor preparation requires 25 h. If all practical steps are taken into account, including repeated washing, reagent handling, pipetting, and final drying, the overall preparation time is approximately 28 h. A similar distinction applies to the analytical assay cycle. The defined incubation steps of the assay require 2 h in total, whereas the complete assay procedure, including all washing, handling, drying, and frequency measurement steps, requires approximately 5 h.

**Figure 2:**
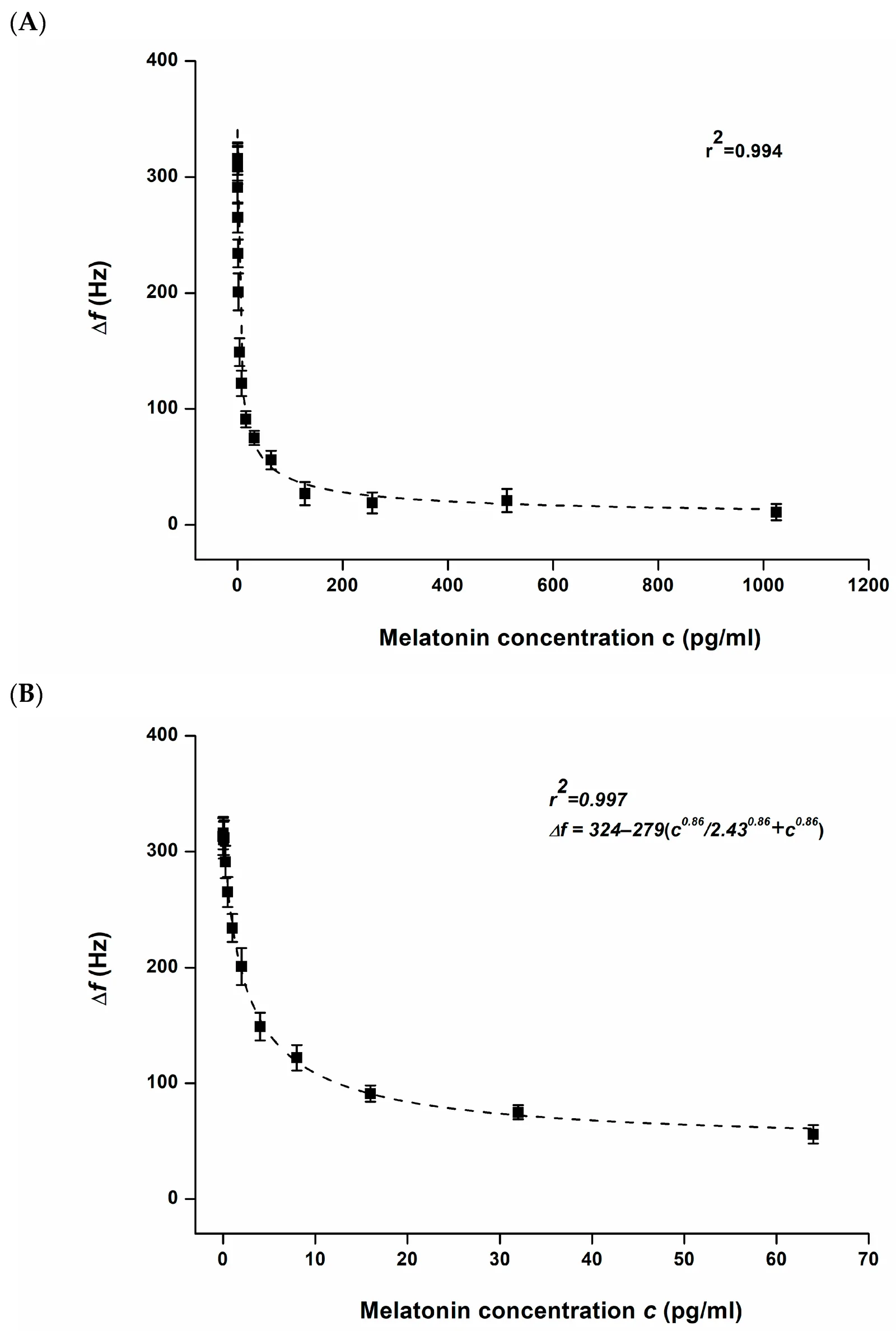
Calibration of QCM biosensor for chemical standard of melatonin. Error bars indicate standard deviation for five repeats. Part (**A**) expresses full range of melatonin assay up to 1024 pg/mL, part (**B**) shows the calibration up to 64 pg/mL.

The prepared QCM biosensor was tested with various concentrations of melatonin in order to evaluate its analytical response, and the obtained results are shown in Figure 2. The calibration curve was constructed from standard melatonin solutions prepared by two-fold serial dilution over the concentration range from 1024 pg/mL to 0.03125 pg/mL. The coefficient of determination was r^2^ = 0.994 for the full calibration range and r^2^ = 0.997 when the calibration was limited to concentrations up to 64 pg/mL (275 pmol/L). The limit of detection was 0.55 pg/mL when estimated from the calibration curve using the criterion of a signal-to-noise ratio equal to 3. Considering molar concentration, the limit of detection is equal to 2.37 pmol/mL. The calibration curve was extrapolated using the Hill equation. The assay did not exhibit linearity over the full calibration range; however, in the range up to 4 pg/mL, the response can be described by an approximately linear dependence with r^2^ = 0.913.

The assay exhibited high sensitivity in the low concentration range, especially up to approximately 10 pg/mL (43 pmol/L), above which the sensitivity gradually decreased. Nevertheless, the assay remains applicable for the concentration levels expected in real samples. Samples containing higher melatonin concentrations can be diluted prior to analysis when necessary. It should also be taken into account that the sample volume used in the assay was 25 µL.

The lower sensitivity observed at concentrations above 64 pg/mL may be a limitation when examining samples with high melatonin content, such as specimens from individuals taking supplementary melatonin or pharmaceutical preparations. Such samples should therefore be preanalytically modified before measurement, for example, via dilution or another suitable sample processing procedure.

The limit of detection achieved by the QCM biosensor is sufficiently low for analysis of melatonin at physiological concentrations. Considering that melatonin occurs in blood at low pg/mL to ng/mL levels and in saliva at even lower concentrations, the detection limit of 0.55 pg/mL remains well below the lowest expected physiological concentration in both matrices. For instance, Lodhi and coworkers stated that the concentration of melatonin in human saliva is in the range 0.8 to 6.8 pg/mL [35]. Blood level reaches higher concentrations. The peak mean concentration in blood of health youth was reported in the range 45 to 54 pg/mL in a study by Inigo and coworkers [36]. The analytical performance compared to physiological concentrations indicates that the biosensor is suitable for determination of melatonin in saliva as well as blood and that it can be used for monitoring endogenous melatonin without the sensitivity becoming a limiting factor.

**Figure 3:**
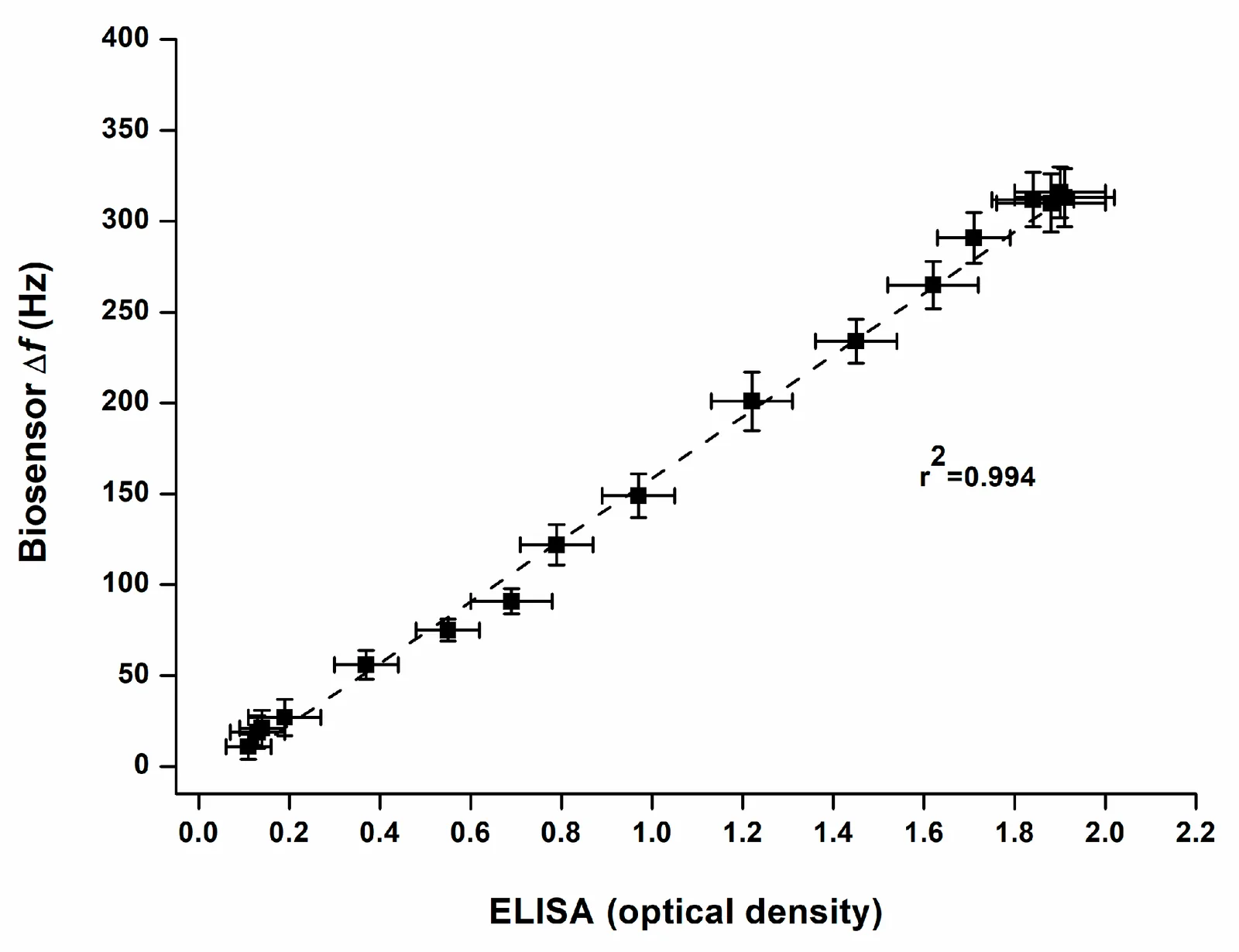
Validation of the QCM biosensor assay for melatonin against the standard ELISA method. A strong correlation was observed between both methods, with a coefficient of determination of r^2^ = 0.994. Each sample was measured five times by the biosensor and five times by ELISA; error bars indicate standard deviation.

The validation experiment (see Figure 3) confirmed very good agreement between the QCM biosensor and the reference ELISA, as documented by the coefficient of determination of r^2^ = 0.994. This result indicates that the newly developed assay provides concentration values fully comparable with the established immunochemical method and that no systematic deviation between the two analytical approaches was apparent within the tested set of samples. The analytical sensitivity was also closely matched, as the biosensor reached the limit of detection described above, while the ELISA provided a limit of detection of 0.61 pg/mL. Both assays further exhibited a similar dynamic range, which supports the practical applicability of the biosensor for samples expected to contain melatonin at physiological levels. A gradual decline in sensitivity was observed at higher melatonin concentrations for both methods, but this effect occurred in a concentration region exceeding the levels normally anticipated in standard biological specimens. From a practical point of view, the parallel performance of both methods supports the use of the QCM biosensor as a reliable alternative to ELISA for melatonin determination.

**Figure 4:**
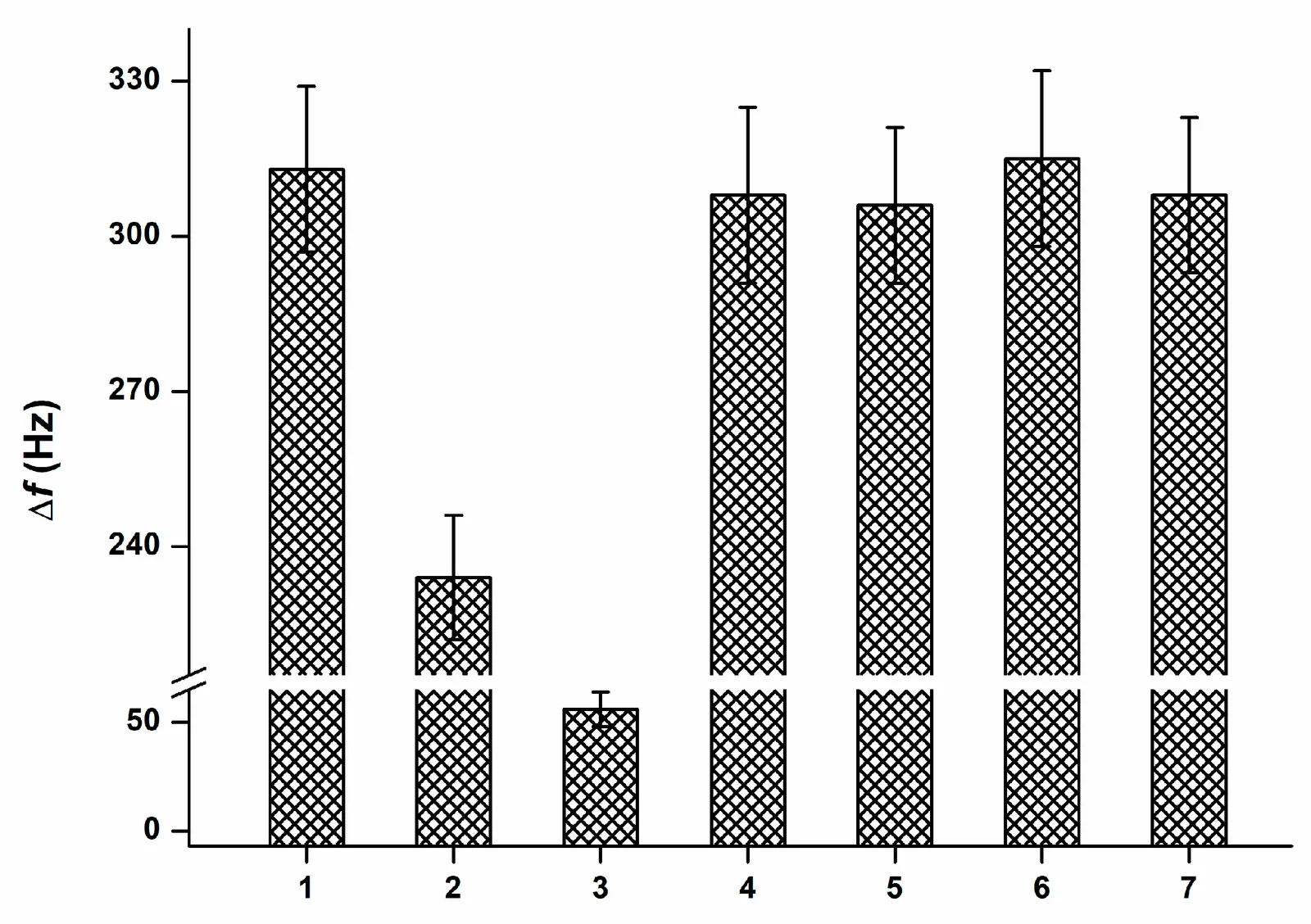
Interference testing. The bars represent the following samples: 1, PBS; 2, melatonin 1 pg/mL; 3, melatonin 64 pg/mL; 4, acetylsalicylic acid 1 mg/mL; 5, caffeine 1 mg/mL; 6, 5-methoxytryptamine 1 ng/mL; 7, tryptamine 1 ng/mL. Each sample was measured five times; error bars indicate the standard deviation.

Potential interference was evaluated using acetylsalicylic acid, caffeine, 5-methoxytryptamine, and tryptamine. These compounds were selected because they may occur in biological samples or are structurally related to melatonin and could therefore affect assay specificity. The tested interferents were compared with PBS as a blank and with melatonin standards at concentrations of 1 pg/mL and 64 pg/mL. The results are presented in Figure 4. The differences in signal, expressed as the change in oscillation frequency (Δ*f*), were insignificant at the ANOVA *p* < 0.01 level when the signal provided by PBS was compared to those caused by 5-methoxytryptamine, caffeine, and tryptamine. By contrast, the signal caused by melatonin at 1 pg/mL differed from the above-mentioned group at the probability level *p* < 0.05 (ANOVA), whereas the concentration of 64 pg/mL differed at *p* < 0.01 (ANOVA). These findings indicate that the biosensor exhibits satisfactory selectivity for melatonin and is not substantially affected by the tested interferents under the applied experimental conditions.

**Figure 5:**
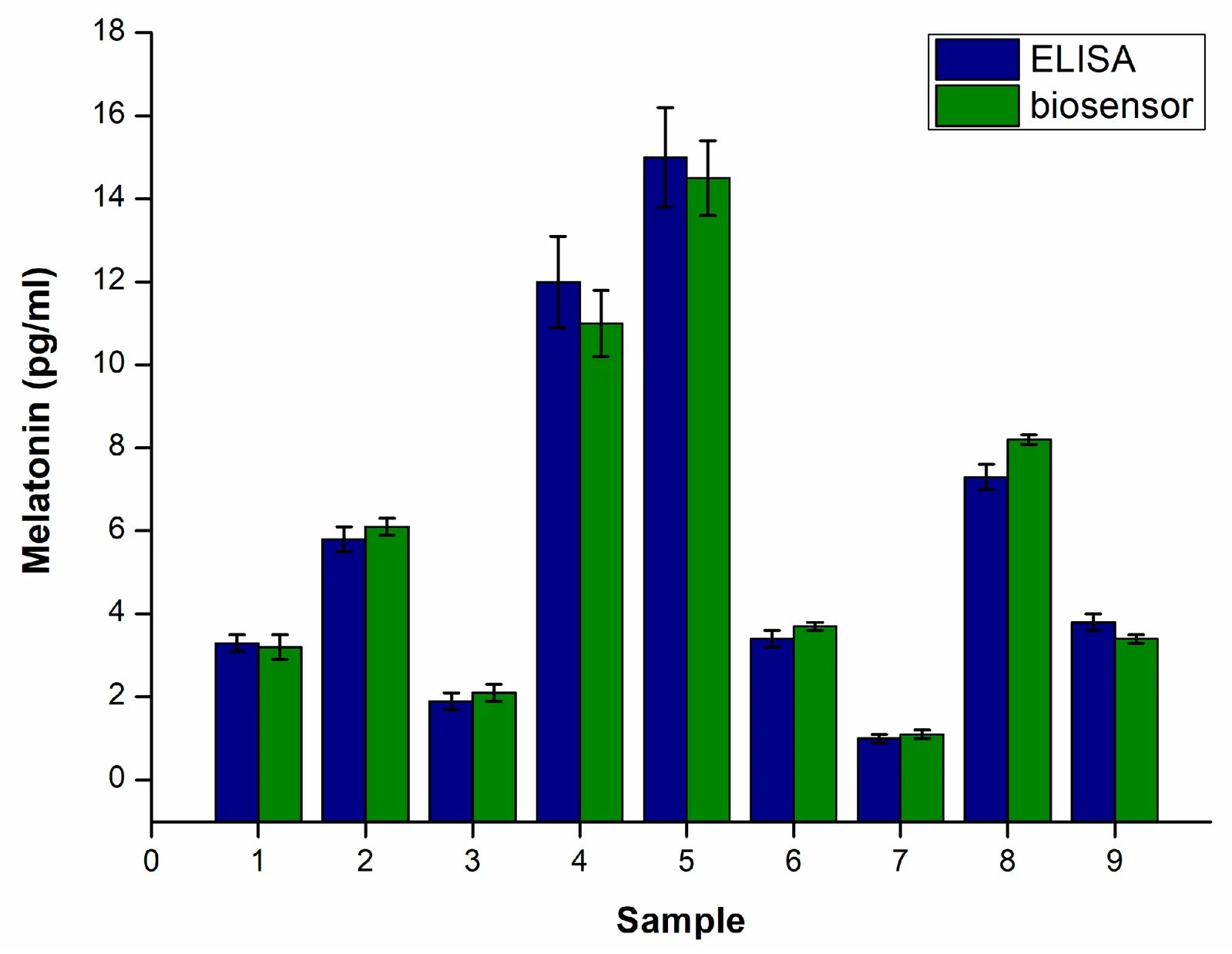
Determination of melatonin in nine authentic human saliva samples collected in the morning and analyzed by the QCM biosensor and reference ELISA method. The measured melatonin concentrations were within the range of 1 to 15 pg/mL. Each sample was analyzed in pentaplicate, and error bars represent standard deviation. Both analytical approaches provided closely matching values, and no significant differences between the measured concentrations were found by ANOVA at *p* < 0.05 or *p* < 0.01.

The experiment performed on real saliva samples confirmed the practical applicability of the proposed assay under routine biological conditions. A total of nine saliva samples obtained in the morning were examined, and the measured melatonin concentrations ranged from 1 to 15 pg/mL, which corresponds well to the expected low abundance of this hormone in saliva. The comparison presented in Figure 5 shows that the QCM biosensor and the ELISA reference assay yielded very similar concentration values throughout the entire set of samples. Statistical evaluation by ANOVA further indicated that the differences between the concentrations found by the two methods were not significant at the tested levels of *p* < 0.05 and *p* < 0.01. These findings support the view that the developed biosensor can provide results comparable to those of the conventional immunoassay when applied to authentic saliva specimens.

This test proved that the matrix effect did not influence the assay results under the applied experimental conditions, and the obtained findings were therefore considered reliable. It should be noted that the saliva samples were kept frozen before analysis, which could have influenced the activity of enzymes naturally present in saliva, especially proteases. Any rearrangement of the experimental protocol, including changes in sample storage or handling, may cause the matrix effect to become significant. Thus, the assay performs well under the current protocol, but this conclusion should not be generalized to substantially modified experimental conditions without further verification.

**Figure 6:**
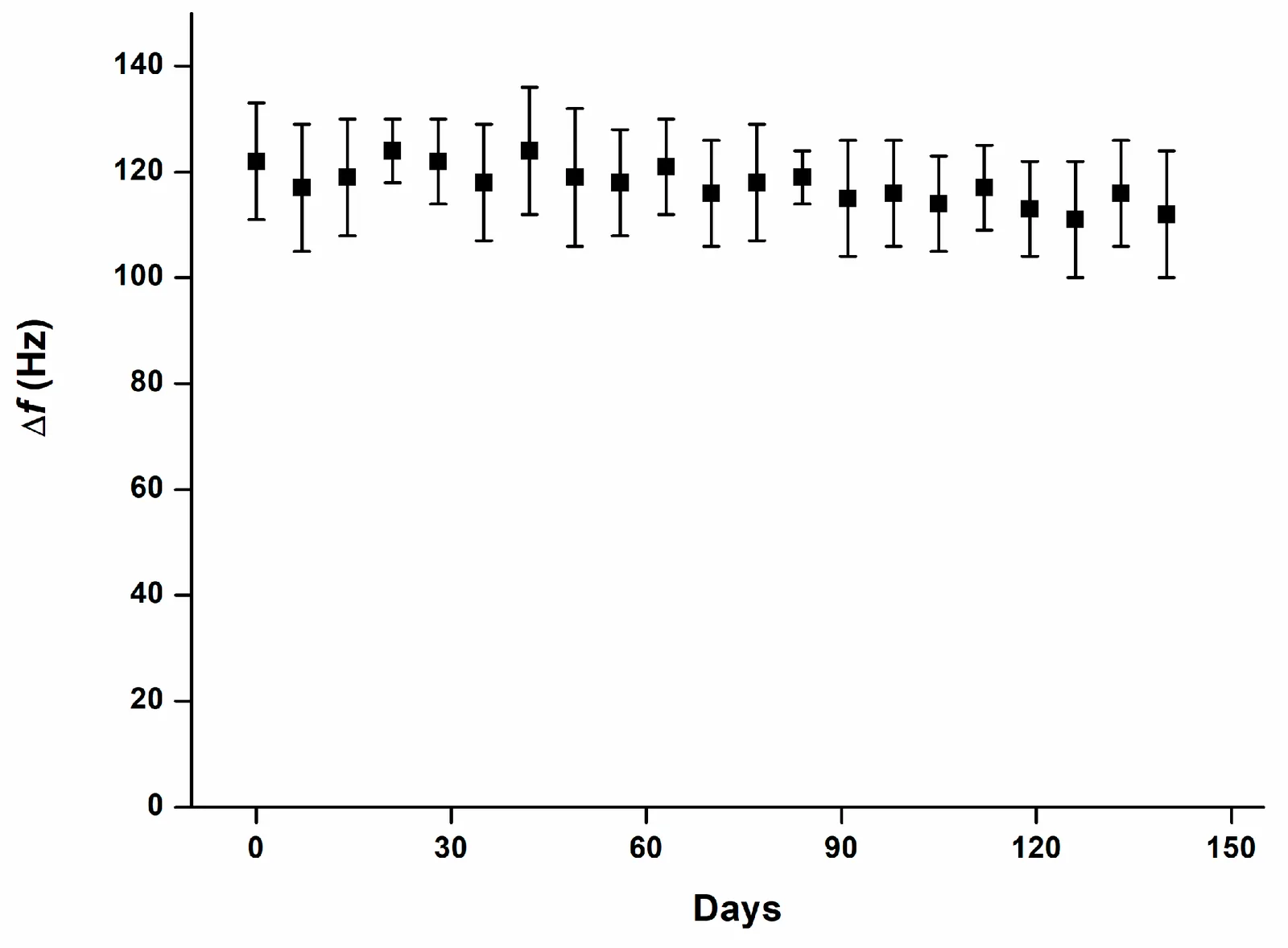
Long-term stability of the biosensor expressed as the signal (Δ*f*) obtained by repeated assay of samples containing 8 ng/mL melatonin. The experiment was performed over 140 days with measurements at 7-day intervals. Each sample was analyzed in pentaplicate, and error bars indicate standard deviation. No significant changes in the recorded signals were observed during the study, and no significant trend was apparent in the measured values over time.

The long-term stability test (see Figure 6) demonstrated that the biosensor maintained a consistent analytical response throughout the full observation period. Repeated measurements of samples containing 8 ng/L melatonin generated comparable Δ*f* values from the beginning of the experiment to day 140, indicating that the sensing surface and assay format remained functionally preserved during storage and repeated verifications. The absence of significant signal changes, together with the lack of any significant temporal trend, suggests that the biosensor does not undergo progressive deterioration that would compromise result reliability within the tested period. This finding is important for practical application because it indicates that the prepared biosensors can be stored and used over an extended time frame without a substantial loss of performance.

The developed QCM biosensor can be compared with several related devices reported in the recent literature. Although the number of biosensors specifically designed for melatonin determination remains limited, several notable examples have been published. Yang et al. developed a polycrystalline boron-doped diamond-based voltammetric biosensor for the simultaneous determination of dopamine and melatonin, with a limit of detection of 0.7 pg/mL (3 pmol/mL) and linear range 93 pg/mL (400 pmol/mL) to 139 pg/mL (600 nmol/mL) for melatonin in an assay lasting 20 to 60 s [37]. Brazaca et al. reported a voltammetric immunosensor for melatonin determination with a limit of detection of 119 pg/mL (513 pmol/mL), linear range 174 pg/mL (750 pmol/L) to 1.7 ng/mL (7.5 nmol/mL), and assay time 1 to 2.5 min [38]. Bisquert et al. described a yeast-based biosensor based on G protein-coupled receptors for melatonin applicable to melatonin assay in fermented beverages. The melatonin was successfully analyzed in the concentration range of 5.1 to 100 pg/mL (22 to 430 pmol/mL) with limit of detection 5.11 pg/mL (22 pmol/mL) and time per one assay 3 to 5 h [39]. These previously published devices were not intended for the determination of melatonin in saliva, and their analytical performance was appropriate for their respective applications. In comparison, the QCM biosensor developed in the present study achieved a lower limit of detection, making it more suitable for analysis of low physiological melatonin concentrations. However, this analytical advantage was obtained through repeated antibody-based signal amplification, which increased assay complexity. Therefore, the proposed format may be less practical in situations where ultra-sensitive detection is not necessary. Furthermore, the assay time required for testing one sample is longer than that reported in the cited papers. Biosensor-based methods represent a promising approach for melatonin determination because they can combine high sensitivity with simpler instrumentation, reduced sample consumption, and potential applicability outside specialized analytical laboratories. Compared with routine assays such as ELISA or chromatographic techniques, biosensors may therefore offer a more flexible platform for rapid or point-of-care testing, although their practical implementation still depends on sufficient validation, reproducibility, and matrix compatibility. Recent progress in this field has been extensively reviewed by Akindele et al. and Duhan et al., who summarized analytical strategies for melatonin detection and highlighted the development of electrochemical, optical, and nanomaterial-based sensing platforms [33,40]. Apart from melatonin, biosensor-based approaches have also been successfully applied to the determination of other hormones. For example, Xu et al. developed an electrochemical biosensor for cortisol detection in body fluids and achieved a very low limit of detection of 0.33 pmol/L, corresponding to approximately 0.12 pg/mL [41]. In another study, Weng et al. prepared a microfluidic origami biosensor for cortisol determination in human sweat, with a reported limit of detection of 6.8 ng/mL [42]. These examples further demonstrate the broader applicability of biosensor platforms for sensitive hormone analysis in biologically relevant matrices. Comparison of the QCM biosensor with other biosensor applications for melatonin assay are depicted in Table 1.

## 4. Conclusions

The constructed QCM biosensor provides a sensitive competitive immunoassay for melatonin and achieves analytical performance suitable for determination of this hormone in physiological concentrations. The low limit of detection, good agreement with the reference ELISA, satisfactory selectivity toward selected interferents, and successful application to authentic saliva samples indicate that the assay can serve as a practical alternative to conventional immunochemical methods. The use of saliva as a specimen is particularly advantageous because it enables non-invasive sampling for circadian rhythm assessment and related biomedical studies.

Beyond the determination of melatonin itself, the presented assay confirms the usefulness of QCM-based competitive immunosensing for low-molecular-weight analytes that are difficult to detect using direct label-free formats. The long-term stability of the prepared biosensors further supports their practical applicability. Although the biosensor was optimized specifically for melatonin, the overall concept based on immobilized analyte conjugate and antibody-mediated signal amplification can be adapted for other small biomarkers where rapid, simple, and sensitive analysis is required.

The biosensor was intentionally constructed to cover very low concentrations of melatonin, which was achieved by three sequential antibody administration steps. This signal amplification strategy enabled the assay to reach the sensitivity required for low physiological concentrations, but it also represents a major drawback because it prolongs the assay protocol and requires the use of three different antibody types. Nevertheless, the approach can be further improved in future studies by incorporating an additional recognition molecule, for example, an antibody conjugate or another suitable recognition element, which could strengthen the analytical response and simplify or enhance the overall assay format. It should also be considered that the presence of several antibody layers may potentially cause assay-related problems, although no such effect was observed in the present experiments.

The developed biosensor can help address the current lack of analytical tools suitable for simple measurement of melatonin in saliva and, respectively, in other biological samples. The obtained results should be considered a proof of concept demonstrating that a QCM-based competitive immunoassay can reach the sensitivity required for physiological melatonin concentrations. Further research may improve this biosensor-based immunoassay approach for melatonin, simplify its analytical protocol, and make it more accessible for routine use outside specialized laboratories or hospital facilities.

## Figures and Tables

**Table 1:** Comparison of biosensors for melatonin determination.

Physical Principle of Biosensing	Bioreceptor Molecule	Limit of Detection	Working Range	Testing Time	References
QCM biosensor working on competitive immunoassay	antibody	0.55 pg/mL	0.55 to 64 pg/mL (up to 1024 pg/mL with reduced sensitivity)	approx. 5 h; (biosensor manufacturing approx. 28 h)	here presented study
polycrystalline boron-doped diamond-based voltammetric biosensor	boron-doped diamond	0.7 pg/mL	linear range 93 pg/mL to 139 pg/mL	20 to 60 s	[37]
voltammetric immunosensor	antibody	119 pg/mL	linear range 174 pg/mL to 1.7 ng/mL	1 to 2.5 min	[38]
yeast-based biosensor	G protein-coupled receptors for melatonin	5.1 to 100 pg/mL	5.11 pg/mL	3 to 5 h	[39]

## Data Availability

All data are presented in the manuscript.
